# Complete chloroplast genomes and comparative analyses of *Hippeastrum ‘*milady’, *Hippeastrum albertii* and *Hippeastrum reticulatum* (Amaryllidaceae)

**DOI:** 10.1371/journal.pone.0271335

**Published:** 2022-08-05

**Authors:** Xiao-fei Liu, Ying-bo Sun, Gen-fa Zhu, Li-li Huang, Bo Yu

**Affiliations:** 1 Environmental Horticulture Institute, Guangdong Academy of Agricultural Sciences, Guangzhou, Guangdong, China; 2 Guangdong Key Lab of Ornamental Plant Germplasm Innovation and Utilization, Key Laboratory of Urban Agriculture in South China, Ministry of Agriculture, Guangzhou, China; Chinese Academy of Medical Sciences and Peking Union Medical College, CHINA

## Abstract

*Hippeastrum* is a genus of ornamental plants with large, brightly colored flowers. Due to the very high seed-setting rate of the hybridization of *Hippeastrum*, the large population of hybrid progeny and the existence of superparent inheritance, it is difficult to trace the origin of the varieties collected from the market during breeding. In this study, we analyzed the chloroplast genomes of *Hippeastrum* ‘Milady’, *H*. *alberti*, and *H*. *reticulatum* using the Illumina NovaSeq sequencing platform and generated full-length sequences of 158,067, 158,067, and 158,522 bp, respectively. All three genomes had the typical tetrad structure. The large single copy, small single copy, and inverted repeat regions of *H*. *reticulatum* were observed to be respectively 277, 138, and 20 bp longer than the corresponding regions of *H*. ‘Milady’ and *H*. *alberti*. The results of comparative analysis of simple sequence repeats (SSRs), Ka/Ks ratios, codon preferences, and complete sequences of chloroplasts of these three taxa and 14 other plant species were as follows. First, the chloroplast genomes of *H*. ‘Milady’, *H*. *alberti*, and *H*. *reticulatum* contain 209, 209, and 211 SSR sites, respectively, most of which (123, 123, and 122, respectively) are single nucleotide repeats. Second, leucine, arginine, and serine are the most frequently used amino acids in the three chloroplast genomes. Third, *H*. ‘Milady’, *H*. *alberti*, and *H*. *reticulatum* are more closely related to *Lycoris* and *Narcissus* than to *Allium* and *Agapanthus*. Our results will provide information on the study of origins or relatedness of native species, and the identification of cultivars.

## Introduction

*Hippeastrum* Herb. (Amaryllidaceae) is a genus of flowering perennials native to South America. The large, brightly colored flowers, which are borne on 50–70-cm long peduncles, can appear at the same time as the leaves. *Hippeastrum* is of high ornamental value, and more than 300 varieties have been developed [1]. In addition, the plants are rich in alkaloids and have thus been investigated for potential anti-anxiety, anti-convulsant, anti-depressive, and anti-toxic properties and for the development of related products [2–4]. Ploidy levels in *Hippeastrum* range from diploid to octoploid [5], and the seed set rate of hybrids is high. Researchers in the Netherlands, the United States, South Africa, and other countries have therefore carried out hybrid breeding work and selected many varieties for use as cut flowers, potted plants, and garden ornamentals [5, 6]. The large number of selected varieties is increasingly hindering the ability to identify and distinguish the original species, hybrid progeny, and cultivars of *Hippeastrum*, which must be performed on the basis of morphology given the lack of genomic data, and therefore it is increasing the difficulty of parental selection in future breeding. Research on the phylogenetic relationships, genetic diversity, and genetic background of *Hippeastrum* and the development of more identification methods and better breeding protocols are thus urgently needed.

Apart from a genetic structural analysis of germplasm resources based on microsatellite markers developed from transcriptome data [5], few studies have been carried out on the genetic structure and phylogeny of *Hippeastrum*. Nuclear, chloroplast, and mitochondrial genomes are widely exploited in phylogenetic analyses. Because of its simple structure, strong conservation, and amenability to sequencing, the chloroplast genome has been used in genetic structural and phylogenetic analyses of a wide range of plant taxa, including *Chlorophytum comosum* [7], Monsteroideae [8], *Hosta* [9], *Aloidendron* [10], *Allium* [11], *Astelia pumila* [12], Agavoideae [13], *Fritillaria ussuriensis* [14], *Amomum kravanh* [15], *Talinum paniculatum* [16], and buckwheat [17]. The chloroplast genomes of various species of Amaryllidaceae have also been published [11, 18–22]. Thus far, however, no research reports have appeared on *Hippeastrum* chloroplast genomes.

In this study, we carried out Illumina sequencing of the chloroplast genomes of *H*. ‘Milady’, *H*. *alberti*, and *H*. *reticulatum* and compared their structural features. Using the sequenced genomes, we also analyzed the phylogenetic relationships of these three taxa with Amaryllidaceae and other monocots. The results of our comparative analysis should lay a foundation for future resource evaluation and phylogenetic analysis of the genus.

## Materials and methods

### Plant materials and extraction of genomic DNA

Genomic DNA was extracted from young leaves (100 mg) of *H*. ‘Milady’, *H*. *alberti*, and *H*. *reticulatum* plants using a plant genomic DNA extraction kit (DP305; Tiangen Biotech, Beijing, China). After mechanical (ultrasound) fragmentation, the extracted DNA was subjected to purification, end repair, poly(A) tail addition, and ligation of sequencing adapters and then analyzed by agarose gel electrophoresis for fragment size selection. PCR amplification was performed to generate a sequencing library, and the qualified library was sequenced on the Illumina NovaSeq platform with a sequencing read length of PE150.

### Genome sequencing, assembly, and annotation

The paired-end Illumina raw reads were filtered using Trimmomatic [23] and then mapped to the chloroplast genome of the reference species *Xanthorrhoea preissi* (GenBank accession no. NC_035996.1), with Bowtie2 v2.2.4 [24] used to exclude reads of nuclear or mitochondrial origin. SPAdes 3.6.1 [25] and Sequencher 5.3.2 (Gene Codes Inc., Ann Arbor, MI, USA) were used for *de novo* assembly to reconstruct the chloroplast genomes. A “genome walking” technique was adopted to remove gaps [26]. Jellyfish v.2.2.3 [27] was used to correct misassembled contigs. CpGAVAS [28] was used for annotation of the chloroplast genomes, and a circular representation was generated with OGDRAW [29].

### Ka/Ks analysis

Base variations resulting in amino acid changes are called non-synonymous mutations, whereas those that do not are termed synonymous mutations. Non-synonymous mutations are generally affected by natural selection. The ratio of the non-synonymous mutation rate (Ka) to the synonymous mutation rate (Ks) indicates the selection effect; a value greater than 1 indicates that a gene is subject to positive selection, while a value less than 1 corresponds to the existence of purifying selection. We aligned gene sequences in mafft v7.310 [30] and then calculated gene Ka/Ks values using KaKs_Calculator v2.0 [31].

### Repeat sequence analysis

The Perl script MISA [32] was used to detect microsatellites (mono-, di-, tri-, tetra-, penta-, and hexanucleotide repeats) with the following unit size and minimum repeat thresholds: 10 repeat units for mononucleotide SSRs, 5 for dinucleotide SSRs, 4 for trinucleotide SSRs, and 3 each for tetra-, penta-, and hexanucleotide SSRs.

### Codon usage and nucleotide diversity

For the identification of codon usage patterns, we used all CDSs present in the three *Hippeastrum* chloroplast genomes to estimate codon usage in CodonW with translational table = 11 [33].

The complete chloroplast genomes of five related species were downloaded from the NCBI database: *Lycoris radiata* (MN158120.1), *Narcissus poeticus* (NC_039825.1), *Agapanthus coddii* (NC_035971.1), *Agave* sp. Pires 2011 (KX931464.1), and *Agave attenuata* (NC_032696.1). The chloroplast genome sequences of these five species and *H*. ‘Milady’, *H*. *alberti*, and *H*. *reticulatum* were first aligned in MAFFT v7 and then manually adjusted using BioEdit software. A sliding window analysis was then conducted to evaluate the nucleotide variability (Pi) of the chloroplast genome using DnaSP v5.1 [34]. The step size was set to 200 bp, and the window length was set to 600 bp.

### IR contraction and expansion

The chloroplast genome is a circular structure, and IR has four boundaries with LSC and SSC, namely LSC-IRb, IRb-SSC, SSC-IRa and IRa-LSC. During genome evolution, IR boundaries expand and contract, allowing certain genes to enter IR regions or single-copy regions. IRscope (https://irscope.shinyapps.io/irapp/) was used for visualizing the genes’ differences on the boundaries of the junction sites of the eight chloroplast genomes: *Hippeastrum* ‘Milady’ (MT162609), *H*. *alberti* (MT701522), *H*. *reticulatum* (MT701523), *Lycoris radiata* (MN158120.1), *Narcissus poeticus* (NC_039825.1), *Agapanthus coddii* (NC_035971.1), *Agave* sp. Pires 2011 (KX931464.1) and *Agave attenuata* (NC_032696.1).

### Phylogenetic analysis

A phylogenetic tree were constructed by the maximum likelihood method using entire chloroplast genomes. The maximum likelihood analysis was performed using RAxML-HPC BlackBox v.8.1.24 [35] at the CIPRES Science Gateway website based on the best-fit model of evolution (GTR + G) with 1,000 bootstrap replicates. The GenBank accession numbers of the analyzed plant genomes are as follows: *Hippeastrum* ‘Milady’ (MT162609), *H*. *alberti* (MT701522), *H*. *reticulatum* (MT701523), *H*. *vittatum* (NC_052724.1), *H*. *rutilum* (MT937175.1), *Lycoris radiata* (MN158120.1), *Narcissus poeticus* (NC_039825.1), *Agapanthus coddii* (NC_035971.1), *Agave* sp. Pires 2011 (KX931464.1), *Agave attenuata* (NC_032696.1), *Allium cepa* (KF728080.1), *Asparagus officinalis* (KY364194.1), *Cordyline indivisa* (KX822776.1), *Milla biflora* (KX822778.1), *Aphyllanthes monspeliensis* (KX790360.1), *Aloe vera* (KX377524.1), *Iris sanguinea* (KT626943.1), *Dendrobium bellatulum* (MG595965.1), *Lilium lankongense* (MK757466.1), *Nomocharis pardanthina* (NC_038193.1), *Tulipa altaica* (NC_044780.1), *Arabidopsis thaliana* (NC_000932.1), and *Celosia cristata* (MK470118.1). The latter two species were used as outgroups.

## Results

### Characteristics of *Hippeastrum* chloroplast sequences

**Fig 1:**
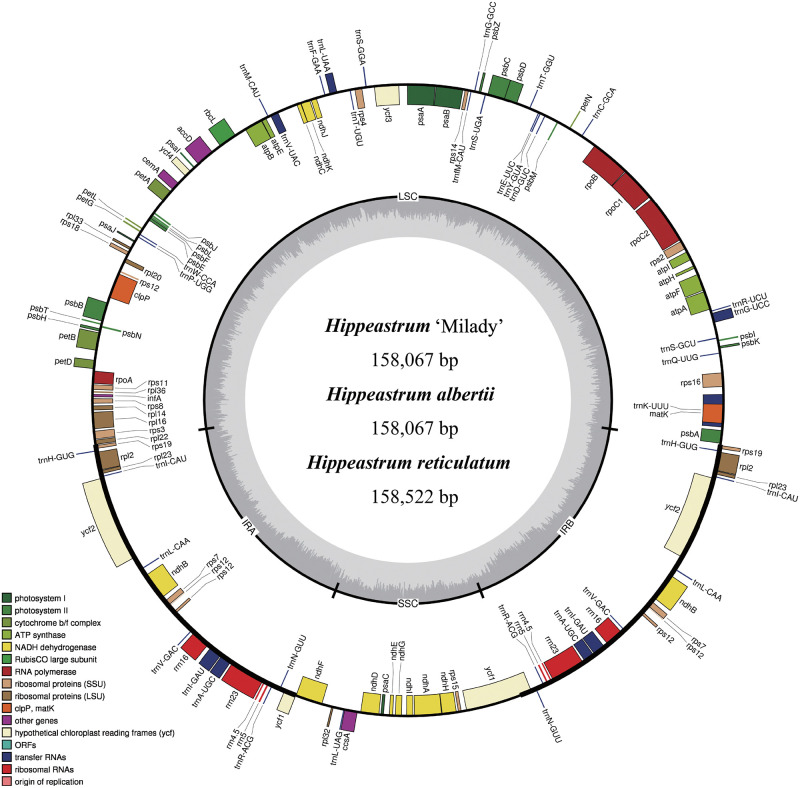
Gene maps of *Hippeastrum* ‘Milady’, *H*. *alberti*, and *H*. *reticulatum*. Genes lying outside the circle are transcribed in a clockwise direction, whereas genes on the inside are transcribed in a counterclockwise direction. Different colors denote known functional groups. The relative GC and AT contents of genomic regions are respectively represented in the inner circle by dark and light gray. LSC, SSC, and IR indicate large single copy, small single copy, and inverted repeat regions, respectively.

The assembled chloroplast genome sequences of *H*. ‘Milady’ (GenBank accession no. MT162609) and *H*. *alberti* (MT701522) were both 158,067 bp, which was 455 bp smaller than that of *H*. *reticulatum* (MT701523; 158,522 bp). All three sequences had the same GC content (37.93%) and the classical quadripartite structure, namely, a large single copy (LSC) region, a small single copy (SSC) region, and a pair of inverted repeat (IR) regions (Table 1 and Fig 1). The shared sequence structure and GC content of these three taxa suggest that the chloroplast genome is highly conserved in the genus *Hippeastrum*. The three *Hippeastrum* chloroplast genomes were all predicted to encode 133 genes: 86 protein-coding genes (PCGs), 38 transfer RNA (tRNA) genes, 8 ribosomal RNA (rRNA) genes, and 1 pseudogene (Table 1).

**Table 1:** Feature of complete chloroplast genomes of *Hippeastrum* ‘Milady’, *H*. *albertii* and *H*. *reticulatum*.

Taxon	Full		LSC		SSC		IR		Gene number	PCGs	tRNAs	rRNAs	pseudo
	Length (bp)	GC (%)	length (bp)	GC (%)	length (bp)	GC (%)	length (bp)	GC (%)					
*H*. ‘Milady’	158,067	37.93	86,166	35.96	18,271	32.2	26,815	43.05	133	86	38	8	1
*H*. *albertii*	158,067	37.93	86,166	35.96	18,271	32.2	26,815	43.05	133	86	38	8	1
*H*. *reticulatum*	158,522	37.93	86,443	35.98	18,409	32.16	26,835	43.05	133	86	38	8	1

### Ka/Ks ratios of species pairs

We compared the Ka/Ks values of 86 PCGs in the chloroplast genomes of *H*. ‘Milady’ or *H*. *alberti* with those of *H*. *reticulatum*. The Ka/Ks ratios of *ndhF* and *ndhD* in the first two taxa were greater than 1, namely, 1.78 and 1.87, respectively (S1 Table), which suggests the occurrence of positive selection along these lineages. In contrast, the Ka/Ks ratios of 15 PCGs were less than 1 (S1 Table), indicative of purifying selection. Six of these genes (*rpoC1*, *psbD*, *ycf3*, *rps4*, *petD*, and *rpoA*) had Ka/Ks ratios ranging from 0.1 to 0.3, which implies strong purifying selection (S1 Table).

### Analysis of sequence repeats

**Fig 2:**
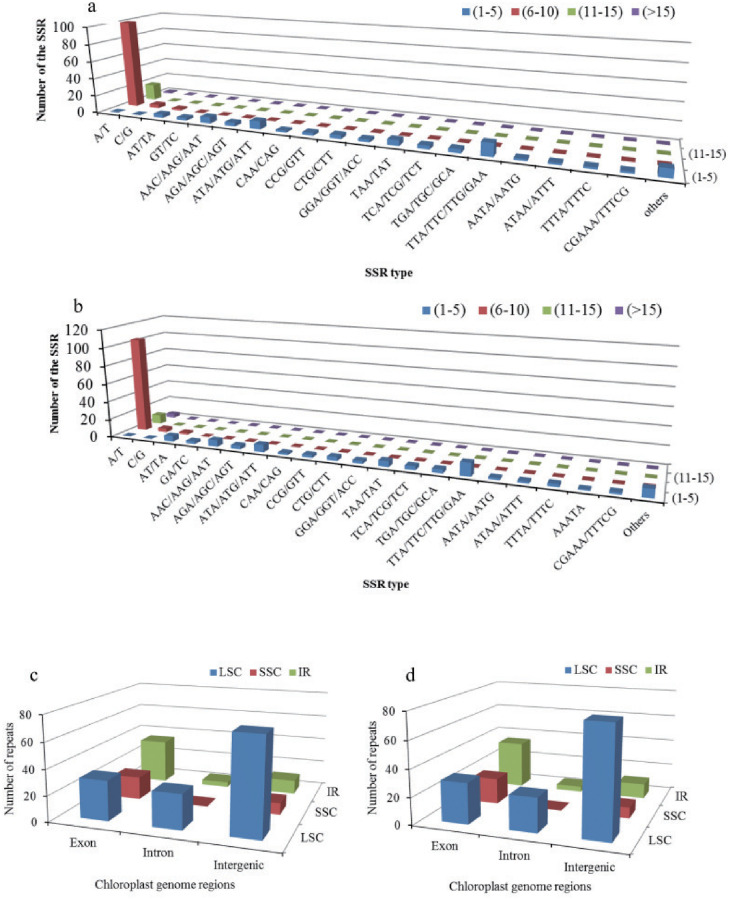
Simple sequence repeats (SSRs) in chloroplast genomes of three *Hippeastrum* taxa. (a–b) SSR types and distributions in *H*. *alberti* (a), *H*. ‘Milady’ (a), and *H*. *reticulatum* (b). (c–d) Distribution of SSRs according to genomic region in *H*. *alberti* (c), *H*. ‘Milady’ (c), and *H*. *reticulatum* (d). LSC, SSC, and IR are large single copy, small single copy, and inverted repeat regions, respectively.

Numerous microsatellites (simple sequence repeats; SSRs) were detected in the three *Hippeastrum* chloroplast genomes, ranging from 209 in *H*. ‘Milady’ and *H*. *alberti* (S2 and S3 Tables) to 211 in *H*. *reticulatum* (S4 Table). The most abundant SSRs were mono-nucleotide repeats, whose proportions relative to the total number of chloroplast genome SSRs were similar among *H*. ‘Milady’, *H*. *alberti*, and *H*. *reticulatum*: 58.9% (123), 58.9% (123), and 57.8% (122), respectively (Fig 2a and 2b). Relative to the total number of SSRs, the proportion of mono-nucleotides repeated 11 to 15 times was quite different among taxa, namely, 8.6%, 8.6%, and 4.7% in *H*. *alberti*, *H*. ‘Milady’, and *H*. *reticulatum*, respectively (Fig 2a and 2b). We also analyzed the distribution of SSRs in different regions of the chloroplast genomes of the three *Hippeastrum* taxa. We found that the number and overall distribution of SSRs in IR regions were the same in the three taxa. In the LSC and SSC regions, the number of SSRs in the chloroplast genomes of the three *Hippeastrum* taxa was slightly different, but their distribution trends were similar (Fig 2c and 2d).

### Codon usage and nucleotide diversity

Codon usage rates vary substantially in the genomes of different species and different organisms. The nonrandom usage of synonymous codons, termed relative synonymous codon usage (RSCU), is considered to be the comprehensive result of natural selection, species mutation, and genetic drift. The three *Hippeastrum* chloroplast genomes all contained 66 codons and a total of 26,390 codons, and their codon usage patterns were also the same. Leucine, arginine, and serine, the most prevalent amino acids, were encoded by the most codons (six), whereas tryptophan was encoded by the fewest. We identified 32 preferred (RSCU > 1.00) and 33 non-preferred (RSCU < 1.00) codons in the three *Hippeastrum* taxa (S5 Table).

Nucleotide diversity values ranged from 0.00000 to 0.04567, with a mean value of 0.01355. Nucleotide diversity ranged from 0.00000 to 0.01837, with an average of 0.0095, in the IR regions, and from 0.0000 to 0.04642, with an average of 0.01372, in the SSC region (S6 Table). Six genes (*ycf1*, *rpl22*, *rps15*, *matK*, *ndhF*, and *ndhD*) had nucleotide diversity values higher than 0.03550, and all except the last two had values above 0.04200. More than 100 mutations were detected in nine genes: *ycf1*, *rpoC2*, *ndhF*, *matK*, *ycf2*, *rpoB*, *ndhD*, *rpoC1*, and *accD* (S6 Table and S1 Fig).

### Global genome alignment and analysis

Analysis of SNPs and insertions/deletions (indels) in the chloroplast genome of *H*. ‘Milady’ or *H*. *alberti* relative to that of *H*. *reticulatum* revealed 328 SNPs in *H*. ‘Milady’ and *H*. *alberti*, including 131 located in protein-coding regions (S7 Table). We detected 87 indels, consisting of 43 insertions and 41 deletions, in the chloroplast genome of *H*. ‘Milady’ or *H*. *alberti* relative to that of *H*. *reticulatum* (S8 Table). Of these 87 indels, 36 (36.08%) were single-base indels, corresponding to 19 insertions and 17 deletions (S8 Table). We used the SNP sites with Id numbers 14/15, 20, and 21 in the S7 Table to design primers as follows by primer3: No 14/15F-TAAGTTCCCATTCACGACCC, No 14/15R-CCCTACCTTATTGACCGCAA, No 20F- CGACCGAATCGATCAAGAAT, No 20R- TTGGTCTCAACCGTACAGGA, and No 21F- TCCTGTACGGTTGAGACCAA, No 21R- TAGGGCCTTCTGGTTCTTCA. After PCR amplification, shanger sequencing was performed, and the sequences of the three species were compared. The results found that the sequences of *H*. ‘Milady’ and *H*. *alberti* are identical, and both of them are different from *H*. *reticulatum* at the SNP site (S2–S4 Figs.).

### IR contraction and expansion

**Fig 3:**
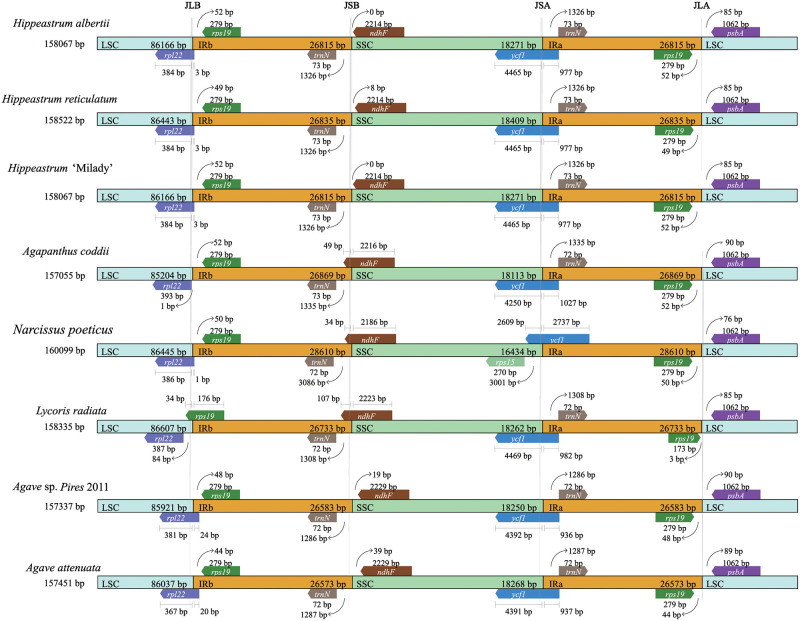
Comparison of the LSC, SSC, and IR regions among eight chloroplast genomes. Boxes above the main line indicate the adjacent border genes. The figure is not to scale with respect to sequence length, and shows only relative changes at or near the IR/SC borders.

Detailed comparisons of the four junctions LSC-IRb (JLB), IRb-SSC (JSB), SSC-Ira (JSA), IRa-LSC (JLA), and among six Amaryllidaceae chloroplast genomes (*Hippeastrum* ‘Milady’, *H*. *alberti*, *H*. *reticulatum*, *Lycoris radiata*, *Narcissus poeticus*, and *Agapanthus coddii*) and two Asparagaceae chloroplast genomes (*Agave* sp. Pires 2011 and *Agave attenuata*) are presented in Fig 3. IR region of three *Hippeastrum* species chloroplast genomes was highly conserved, and slightly structure variation were found in the JLB and JSB regions (Fig 3). The *rpl22*-*rps19* gene were located at the junctions of the JLB regions in *H*. ‘Milady’, *H*. *alberti*, *H*. *reticulatum*, *L*. *radiata*, *N*. *poeticus*, *A*. *coddii*, and *A*. sp. Pires 2011, and *A*. *attenuate*, and the *rps19* gene is located in the IRb region 52, 49, and 52 bp away from the JLB border in three *Hippeastrum* chloroplast genomes, respectively (Fig 3). The *trnN*-*ndhF* genes were located in the junctions of the JSB regions. The *trnN* gene is located in the IRb region ~1300 bp away from the JSB border in eight species except *N*. *poeticus* (3086 bp) (Fig 3). The *ycf1* gene was located in the junctions of the JSA regions in the eight species (Fig 3). However, the distance (2609 bp) from the JSA boundary in the part of the *ycf1* gene located in the SSC region in the chloroplast genome of *N*. *poeticus* as significantly different (about 4400 bp) from the other seven species (Fig 3). The *rps19*-*psbA* genes were located in the junctions of JLA regions, and the *rps19* gene was located in the IRa region 3 bp away from the JLB border in *L*. *radiate* different from the other 7 species (Fig 3).

### Phylogenetic analysis

**Fig 4:**
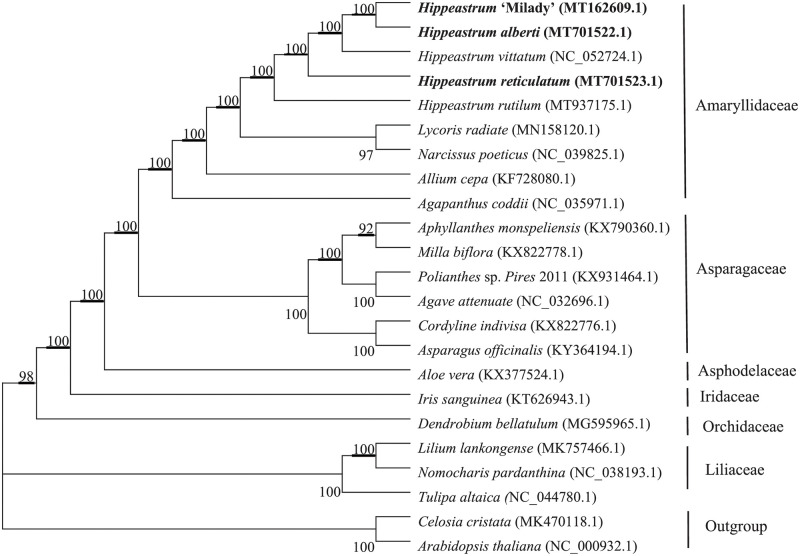
Phylogenetic tree based on maximum likelihood analysis of sequences from whole chloroplast genomes of 18 plant species. *Arabidopsis thaliana* and *Celosia cristata* were used as outgroups. Numbers at nodes are bootstrap support percentages.

To analyze phylogenetic relationships of *Hippeastrum*, we generated a multiple alignment of full-length sequences of chloroplast genomes of *H*. ‘Milady’, *H*. *alberti*, *H*. *reticulatum*, and 18 other monocots plus two outgroups (*Arabidopsis thaliana* and *Celosia cristata*) and carried out a maximum likelihood analysis. In the resulting tree shown in Fig 4, *H*. *alberti* and *H*. ‘Milady’, which have identical chloroplast genome sequences, are clustered together, and the five genomes of *Hippeastrum* form a distinct clade (Fig 4). All nine analyzed species in Amaryllidaceae group together, and *H*. ‘Milady’, *H*. *alberti*, and *H*. *reticulatum* are more closely related to *H*. *vittatum* than to *H*. *rutilum*. According to the tree, *H*. ‘Milady’, *H*. *alberti*, and *H*. *reticulatum* are more closely related to *Lycoris radiata* and *Narcissus poeticus* than to *Allium cepa* and *Agapanthus coddii* (Fig 4). The phylogenetic relationships of Amaryllidaceae to Asparagaceae, Asphodelaceae, Iridaceae, and Orchidaceae revealed in the tree should provide a theoretical basis for future evolutionary analyses of Amaryllidaceae species.

## Discussion

In this study, the complete chloroplast genomes of *H*. ‘Milady’, *H*. *alberti*, and *H*. *reticulatum* obtained by Illumina sequencing ranged from 158.1 to 158.5 kb in length. Comparative analysis revealed that the number of PCGs and tRNAS in the three sequenced genomes is similar to that of other species in Amaryllidaceae, including *Lycoris* (87 PCGs) [22] and *Allium* (81–87 PCGs) [36], but exceeds that of *Celosia cristata* (73 PCGs) [37]. The number of rRNAs in the chloroplast genomes, eight, is conserved in Amaryllidaceae.

Our analysis of Ka/Ks ratios uncovered two genes undergoing positive selection in *H*. ‘Milady’/*H*. *alberti* relative to *H*. *reticulatum*: *ndhD* and *ndhF*, both of which are single-copy genes located in the SSC region (Fig 1). We also selected *Lycoris radiata*, *Narcissus poeticus*, and *Agapanthus coddii* from Amaryllidaceae as well as *Agave* sp. Pires 2011 and *Agave attenuata* from Asparagaceae for a Ka/Ks ratio pairwise analysis. The results of this analysis suggest that the number of positively selected genes between species in different families was greater than that between species in the same family (S9–S18 Tables). These findings are in agreement with the results of previous research on *Chrysosplenium* showing lower Ka/Ks ratios at the chloroplast genome level within *Chrysosplenium* compared with non-*Chrysosplenium* species [38], but they differ from the outcome of a study in *Allium* in which higher pairwise Ka/Ks ratios were observed in *Allium* (Allioideae) species pairs than in non-Allioideae species pairs [11]. Purifying selection constantly sweeps away deleterious mutations in a population. The lower number of positively selected chloroplast genes in Amaryllidaceae compared with Asparagaceae may be the evolutionary result of the preservation of the adaptive characteristics of Amaryllidaceae species.

The GC content of plant species ranged from 19.5% to 42.1% [39]. In this study, we observed a lower GC content in the chloroplast genomes of *H*. ‘Milady’, *H*. *alberti*, and *H*. *reticulatum*, which is similar to *Lycoris* [22] and *Allium* [36]. AT-mutation pressure or AT-biased gene conversion translational efficiency may lead to the paucity of G and C nucleotides observed in plastid genomes [40, 41]. Because they have three hydrogen bonds, GC pairs are more stable than AT pairs, which have only two hydrogen bonds [42].

Expansion and contraction at the junction regions of IRs are the main explanations for the size variation and are common evolutionary events among chloroplast genomes in different species [43–45]. In this study, we compared three *Hippeastrum* species with two species of other genus of Amaryllidaceae and two species of Asparagaceae using chloroplast genomes. It was found that the expansion of IR regions resulted in the IR regions of *Narcissus poeticus* being more than a kilo base longer than the other seven species sequences selected. At the same time, we also found that the distance between *rps19* gene and JLA was shortened in JLA regions. These various regions in the chloroplast genome may provide us with more information in genetic structural analysis. Our phylogenetic analysis clearly revealed that 6 species of Amaryllidaceae clustered well into one clade and 5 *Hippeastrum* species clustered well into one clade.

## Supporting information

S1 Fig The nucleotide diversity value of all genes in *Hippeastrum ‘*Milady’, *H*. *albertii*, *H*. *reticulatum*, *Lycoris radiate*, *Narcissus poeticus*, *Agapanthus coddii*, *Agave* sp. *Pires* 2011 and Agave attenuate. (EPS)

S2 Fig Sequence alignment results after PCR amplification and sequencing of primer No 14/15. (TIF)

S3 Fig Sequence alignment results after PCR amplification and sequencing of primer No 20. (TIF)

S4 Fig Sequence alignment results after PCR amplification and sequencing of primer No 21. (TIF)

S1 Table Ka/Ks ratios of the chloroplast genomes between *H*. ‘Milady’/*H*. *albertii* and *H*. *reticulatum*. (XLSX)

S2 Table Information about SSRs in the chloroplast genome of *Hippeastrum* ‘Milady’. (XLSX)

S3 Table Information about SSRs in the chloroplast genome of *Hippeastrum albertii*. (XLSX)

S4 Table Information about SSRs in the chloroplast genome of *Hippeastrum reticulatum*. (XLSX)

S5 Table Statistical results of relative synonymous codon usage in the chloroplast genome of *H*. ‘Milady’, *H*. *albertii* and *H*. *reticulatum*. (XLT)

S6 Table Statistical results of the nucleotide diversity value. (XLSX)

S7 Table SNPs in the chloroplast genome of *H*. ‘Milady’ or *H*. *albertii* relative to that of *H*. *reticulatum*. (XLSX)

S8 Table Indels in the chloroplast genome of *H*. ‘Milady’ or *H*. *albertii* relative to that of *H*. *reticulatum*. (XLSX)

S9 Table Ka/Ks ratios of the chloroplast genomes between *H*. ‘Milady’/*H*. *albertii* and *Agave* sp. *Pires* 2011. (XLSX)

S10 Table Ka/Ks ratios of the chloroplast genomes between *H*. ‘Milady’/*H*. *albertii* and *Lycoris radiate*. (XLSX)

S11 Table Ka/Ks ratios of the chloroplast genomes between *H*. ‘Milady’/*H*. *albertii* and *Agave attenuate*. (XLSX)

S12 Table Ka/Ks ratios of the chloroplast genomes between *H*. ‘Milady’/*H*. *albertii* and *Agapanthus coddii*. (XLSX)

S13 Table Ka/Ks ratios of the chloroplast genomes between *H*. ‘Milady’/*H*. *albertii* and *Narcissus poeticus*. (XLSX)

S14 Table Ka/Ks ratios of the chloroplast genomes between *H*. *reticulatum* and *Agave* sp. *Pires* 2011. (XLSX)

S15 Table Ka/Ks ratios of the chloroplast genomes between *H*. *reticulatum* and *Lycoris radiate*. (XLSX)

S16 Table Ka/Ks ratios of the chloroplast genomes between *H*. *reticulatum* and *Agave attenuate*. (XLSX)

S17 Table Ka/Ks ratios of the chloroplast genomes between *H*. *reticulatum* and *Agapanthus coddii*. (XLSX)

S18 Table Ka/Ks ratios of the chloroplast genomes between *H*. *reticulatum* and *Narcissus poeticus*. (XLSX)
