# Supplementary figures and images for: Complete chloroplast genomes and comparative analyses of Hippeastrum ‘milady’, Hippeastrum albertii and Hippeastrum reticulatum (Amaryllidaceae)
Source: PLoS One. 2022 Aug 5;17(8):e0271335. doi: 10.1371/journal.pone.0271335 (PMC9355175; doi:10.1371/journal.pone.0271335)

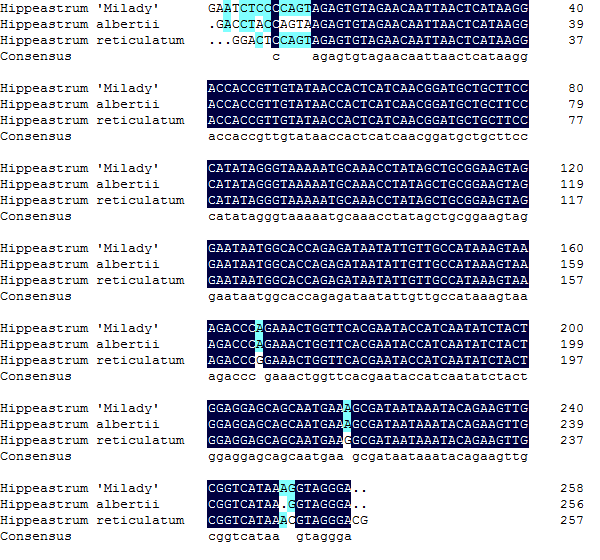

Supplement: S2 Fig — (TIF) [file pone.0271335.s002.tif]

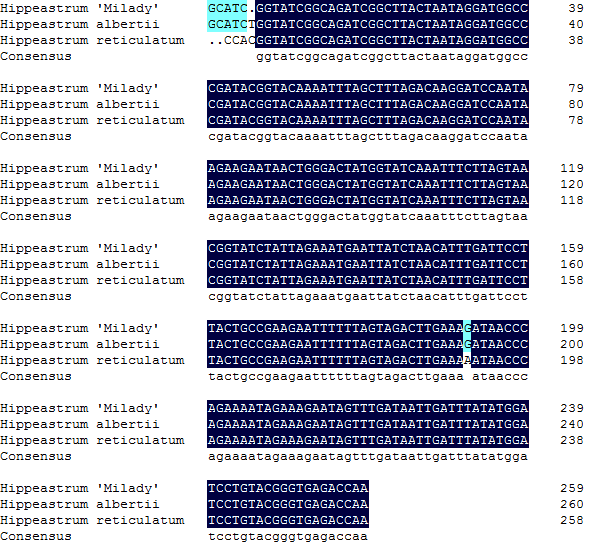

Supplement: S3 Fig — (TIF) [file pone.0271335.s003.tif]

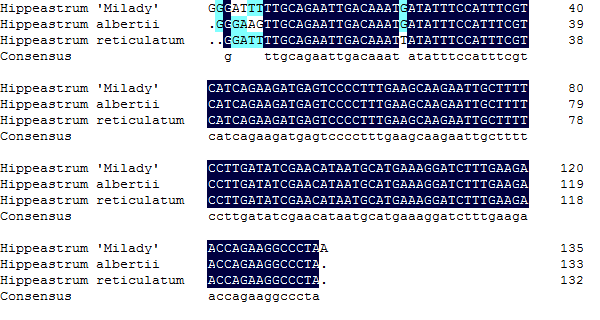

Supplement: S4 Fig — (TIF) [file pone.0271335.s004.tif]
